# Pol IV-Dependent siRNA Production is Reduced in *Brassica rapa*

**DOI:** 10.3390/biology2041210

**Published:** 2013-09-30

**Authors:** Yi Huang, Timmy Kendall, Rebecca A. Mosher

**Affiliations:** 1 School of Plant Sciences, University of Arizona, Tucson, AZ 85721, USA; E-Mails: yih@email.arizona.edu (Y.H.); kendallt@email.arizona.edu (T.K.); 2 Bio5 Institute, University of Arizona, Tucson, AZ 85721, USA

**Keywords:** *Brassica rapa*, RNA Polymerase IV, RNA-directed DNA methylation, siRNA

## Abstract

Plants produce a diverse array of small RNA molecules capable of gene regulation, including Pol IV-dependent short interfering (p4-si)RNAs that trigger transcriptional gene silencing. Small RNA transcriptomes are available for many plant species, but mutations affecting the synthesis of Pol IV-dependent siRNAs are characterized only in *Arabidopsis* and maize, leading to assumptions regarding nature of p4-siRNAs in all other species. We have identified a mutation in the largest subunit of Pol IV, *NRPD1*, that impacts Pol IV activity in *Brassica rapa*, an agriculturally important relative of the reference plant *Arabidopsis*. Using this mutation we characterized the Pol IV-dependent and Pol IV-independent small RNA populations in *B. rapa*. In addition, our analysis demonstrates reduced production of p4-siRNAs in *B. rapa* relative to *Arabidopsis*. *B. rapa* genomic regions are less likely to generate p4-siRNAs than *Arabidopsis* but more likely to generate Pol IV-independent siRNAs, including 24 nt RNAs mapping to transposable elements. These observations underscore the diversity of small RNAs produced by plants and highlight the importance of genetic studies during small RNA analysis.

## 1. Introduction

Most eukaryotes produce an array of small (20–30 nt) RNA molecules (sRNA) capable of gene regulation. In plants the primary classes of sRNAs include 21 nt micro (mi)RNAs and 24 nt short interfering (si)RNAs. MicroRNAs regulate gene expression post-transcriptionally through mRNA degradation or sequestration [1] while 24 nt siRNAs affect gene expression transcriptionally through chromatin modifications that inhibit Pol II transcription or through co-transcriptional degradation of nascent transcripts [2]. 

In the reference plant *Arabidopsis thaliana*, biosynthesis of 24 nt siRNAs is initiated by the plant-specific DNA-dependent RNA polymerase Pol IV [3], which generates a single-stranded RNA template for the RNA-dependent RNA polymerase RDR2 [4]. Double-stranded (ds)RNA produced by RDR2 is cleaved by the DICER-LIKE endonuclease DCL3 into 24 nt duplexes [5]. These duplexes are integrated into ARGONAUTE (AGO) proteins, which are recruited to specific genomic regions through Watson-Crick base pairing between the p4-siRNA and nascent transcripts generated by a second plant-specific DNA-dependent RNA polymerase, Pol V [6]. AGO/p4-siRNA/Pol V complexes enlists a number of proteins, including the DNA methyltransferase DOMAINS REARRANGED METHYLTRANSFERASE 2 (DRM2), to initiate RNA-directed DNA methylation and transcriptional silencing [7]. 

Pol IV-dependent (p4-)siRNAs are the largest class of *Arabidopsis* sRNAs—comprising 75% of the mass and over 99% of the complexity (unique sRNA sequences) in the sRNA transcriptome. P4-siRNAs are produced from thousands of genomic loci, compared to hundreds for all other classes of sRNA combined [3,8,9]. Most p4-siRNAs are produced from repetitive genomic elements [3,8,9] including transposable elements (TEs) [5,10,11,12,13]. P4-siRNAs are also required for *de novo* methylation of incoming transgenes [14] or new retrotransposon insertions [15,16], leading to the hypothesis that p4-siRNAs defend the genome against mobile genetic elements [2,17,18]. 

Small RNA sequencing from many species [19,20,21,22,23,24,25,26] indicates that 24 nt sRNAs are the most abundant class of sRNA in flowering plants, and these sRNAs frequently match TE sequences. However, because DCL3 also acts on dsRNA generated from Pol II-derived RNA hairpins [9,27,28,29] and dsRNA from other pathways [16], it is not possible to determine if these 24 nt sRNAs are Pol IV-dependent without loss-of-function Pol IV mutants. Currently, mutations affecting Pol IV activity are characterized only in *Arabidopsis* and maize. In maize, mutation of Pol IV and downstream components causes loss of 24 nt siRNAs [19,30,31]. However unlike *Arabidopsis*, 22 nt siRNAs of unknown function continue to be produced from many TE sequences when the maize Pol IV pathway is disrupted [19], suggesting that multiple sRNA pathways target TEs. Maize and *Arabidopsis* also differ in the developmental phenotypes associated with loss of Pol IV activity. Maize mutants display abnormal morphology [30] while *Arabidopsis* mutants are indistinguishable from wild type. With Pol IV mutants lacking in other species, generalizations regarding the synthesis of 24 nt siRNAs and the control of TEs cannot be drawn.

*Arabidopsis* is a member of the *Brassicacea* family, which also includes important oil and vegetable species in the *Brassica* genus such as *Brassica oleracea* (broccoli, cauliflower, cabbage, kohl rabi, kale, and brussel sprouts), *Brassica rapa* (turnip, pak choi, Chinese cabbage, and yellow sarsen), *Brassica juncea* (mustard), and *Brassica napus* (canola) [32]. Recently a number of genetic and genomic tools were created to study these important species. In particular, the ~280 Mb genome of *B. rapa* was sequenced [33] and a large *B. rapa* TILLING (Targeting Induced Local Lesions IN Genomes) population created [34]. Within this extensively-mutated population of nearly 10,000 lines, approximately half of the GC base-pairs in the genome are mutated, making possible the selection of both loss-of-function and hypomorphic alleles. Small RNA transcriptomes were analyzed in *Brassica napus*, but Pol IV-dependent siRNAs could not be identified [26]. To further understand the role of Pol IV in production of siRNAs, we characterized sRNA populations from *B. rapa* mutants lacking functional Pol IV.

## 2. Experimental Section

*B. rapa* orthologs were identified through BLASTP search of the Brassica database [35] using *Arabidopsis* protein sequences (TAIR version 10) and confirmed through synteny analysis. The target region was selected based on output of the CoDDLe program (Codons to Optimized Discovery of Deleterious LEsions) and screening of the TILLING population occurred at RevGenUK (Norwich, UK). Putative *brnrpd1* mutations were confirmed and homozygous mutants selected from segregating populations using CAPS/dCAPS markers (see Supplementary Figure S1). For *brnrpd1-1*, a PCR product was amplified with BrNRPD1.F1 (AAACGGGAGACAGCTTCTTACG) and BrNRPD1.R2 (TCAGAGGGTA-AGCGTACTCG) and cleaved with Mbo1 resulting in 400 and 100 nt bands in wild type and 250, 150, and 100 nt bands in *brnrpd1-1*. For *brnrpd1-2*, a PCR product was amplified with BrNRPD1.F1 and BrNRPD1.R1 (AGGAACGTACCCGTGAAGACAGACT) and cleaved with HinF1, resulting in 175 and 25 nt bands in wild type and an uncleaved 200 nt band in *brnrpd1-2* (see Supplementary Figure S1).

Total nucleic acid from a mixture of floral and young silique tissue was prepared as described previously [36] and checked for integrity on a 1% agarose gel. Genomic DNA was removed with Ambion DNA-free according to manufacturer’s instructions before submission to the University of Missouri Genomics Core for library preparation and sequencing.

After trimming adapters and parsing multiplexed data, sRNA reads were filtered to retain high quality reads of appropriate size (19–26 nt) and to remove reads matching the sense strand of structural RNAs (rRNA, tRNA, snRNA, and snoRNA). Reads were then aligned to the *B. rapa* genome using Bowtie (version 0.12.7) [37] and only reads with a perfect genomic match were retained for analysis. Because miRNAs and other 21 nt sRNA species are unlikely to be affected by mutation in p4-siRNA biosynthesis [3,5,8], size profiles of libraries were normalized to filtered, genome-matching 21 nt reads. For all other analyses, libraries were normalized to all filtered, genome-matching reads. Annotation of the *B. rapa* genome was as in [33]. Z scores were calculated by collecting annotation at randomly selected windows from the *B. rapa* genome (100 iterations).

## 3. Results and Discussion

### 3.1. The Pol IV Pathway in *Brassica Rapa*

To analyze the synthesis of sRNAs in *Brassica* species, mutations were sought in p4-siRNA biosynthesis and function genes. The *B. rapa* genome underwent a whole genome triplication event 5–9 million years ago and many genes are still found in triplicate [33]. Homology searches of available *B. rapa* genome sequence revealed a single gene (Bra027611) with 78% amino acid identity to *Arabidopsis NRPD1*. Subsequent analysis of the completed *B. rapa* genome at the Brassica database [35] confirmed that this gene is syntenic to *AtNRPD1* and paralogs do not exist in *B. rapa*, therefore we refer to this gene as *BrNPRD1*. Similar analysis of other Pol IV components and associated proteins reveals that nearly all of these genes reduced to single copy after the genome triplication (Supplementary Table S1). 47% of *Arabidopsis* genes have more than one syntenic ortholog in *B. rapa* [33] and the gene dosage hypothesis [38] suggests that genes which physically interact in complexes are less likely to reduce copy number following whole genome duplication. Consistent with this, components of RNA polymerase II remain in multiple copies in the *B. rapa* genome. However, genes involved in other sRNA pathways, including those not known to function in complexes, such as DCLs, AGOs, and RDRs, also generally reduced to a single copy, suggesting that RNA silencing components might be susceptible to gene loss (Supplementary Table S1). 

**Figure 1:**
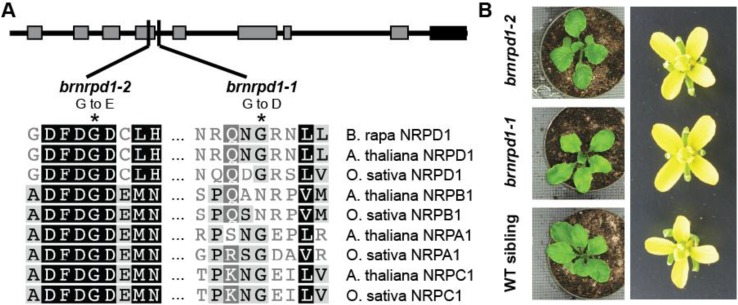
Mutations in *Brassica rapa NRPD1*. (**a**) Location and nature of two missense mutations in Bra027611, BrNRPD1. The protein schematic depicts the eight conserved polymerase regions (gray boxes) and the DeCL domain (black box). Alignment shows conservation of the residues in *Arabidopsis* and rice DNA-dependent RNA polymerases (NRPB1 = Pol II, NRPA1 = Pol I, NRPC1 = Pol III). (**b**) Morphology of *brnrpd1-1* and *brnrpd1-2* leaves and flowers compared to wild type (R-o-18).

To identify a *B. rapa* mutant lacking Pol IV-dependent siRNAs, a *B. rapa* TILLING population [34] was screened for potential loss-of-function mutations in *BrNRPD1*. Within the target region eleven mutations were discovered; four silent mutations and five missense mutations in non-conserved residues were discarded. The remaining two mutations change amino acids that are highly conserved among RNA polymerase largest subunits (Figure 1A). These mutations were designated *brnprd1-1* and *brnrpd1-2* and homozygous individuals were identified for subsequent analysis. The *brnrpd1-1* mutation is just outside the conserved D region, and the *brnrpd1-2* mutation is in the highly conserved Metal A binding site [39]. As in *Arabidopsis*, homozygous *brnrpd1* individuals are phenotypically indistinguishable from wild type or heterozygous siblings (Figure 1B).

### 3.2. Small RNA Production in *Brassica Rapa*

To characterize the *B. rapa* small RNA population, sRNA-seq libraries were generated from R-o-18 (wild type), *brnrpd1-1*, and *brnrpd1-2* reproductive tissue and sequenced on the Illumina platform. The resulting reads were filtered and matched to the genome before analysis (Table 1). Identical filters were applied to published *Arabidopsis* Columbia-0 and *atnrpd1-3* datasets [40] as a control. Although the R-o-18 strain differs from the sequenced reference strain, the percentage of sRNAs matching the genome is not appreciably different between *B. rapa* and *Arabidopsis* libraries, suggesting few sRNAs were lost due to overlap with SNPs.

**Table 1:** Small RNA-seq libraries.

Dataset	Total reads	Filtered		Genome matching		Non-redundant	
R-o-18 #1	6,382,869	4,188,806	65.63%	2,194,063	52.38%	544,690	24.83%
R-o-18 #2	10,801,569	7,019,933	64.99%	3,781,636	53.87%	708,041	18.72%
*brnrpd1-1* #1	11,043,788	7,183,286	65.04%	3,860,502	53.74%	943,725	24.45%
*brnrpd1-1* #2	11,103,304	7,265,692	65.44%	3,837,612	52.82%	774,524	20.18%
*brnrpd1-2* #1	6,967,097	4,676,438	67.12%	2,290,659	48.98%	481,686	21.03%
*brnrpd1-2* #2	10,687,391	7,233,704	67.68%	3,453,687	47.74%	553,872	16.04%
Columbia	11,777,408	6,452,321	54.79%	3,879,919	60.13%	1,525,519	39.32%
*atnrpd1-3*	10,531,269	3,873,131	36.78%	1,830,698	47.27%	365,031	19.94%

**Figure 2:**
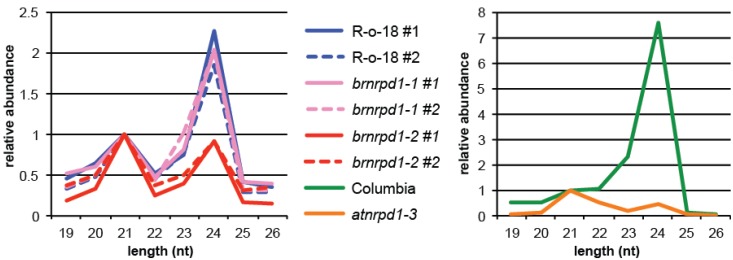
Size profile of small RNAs. Filtered, genome-matching sRNA reads from reproductive tissue of *B. rapa* (**left**) and *Arabidopsis* (**right**). R-o-18 and Columbia are wild type. Biological replicates are denoted #1 and #2.

Length of the sRNA is an important factor in determining sRNA function, because it determines which Argonaute effector protein the sRNA will direct. In *Arabidopsis* AGO1, AGO2, AGO7, and AGO10 prefer to bind 21 nt sRNAs, which are predominantly microRNAs triggering post-transcriptional silencing [41]. In contrast, AGO4, AGO6, and AGO9 associate primarily with 24 nt sRNAs and target nascent RNAs to induce DNA methylation [41,42]. In *B. rapa*, the most abundant sRNA sizes are 24 nt and 21 nt, consistent with other sequenced plant small RNA transcriptomes [19,20,21,22,23,24,25,26]. Unlike its close relative *B. napus*, 22 and 23 nt sRNAs are infrequent in *B. rapa* [26]. Twenty-four nt sRNA are approximately 2-fold more abundant than 21 nt siRNAs, whereas this ratio is 7.5 to 1 in *Arabidopsis* (Figure 2). MiRNAs comprise a higher proportion of 21mers in *B. rapa* than *Arabidopsis* (44% *versus* 22%), suggesting that the reduced ratio of 24:21 nt sRNAs is not due to expression of additional 21 nt siRNAs in *B. rapa*, but rather decreased expression of 24 nt siRNAs relative to *Arabidopsis*. Loss of Pol IV activity through the *atnrpd1-3* mutation reduces the number of 24 nt siRNAs by over 15-fold, reflecting the importance of Pol IV in synthesis of 24 nt siRNAs in *Arabidopsis* (Figure 2). In *brnrpd1-2* reduction in 24 nt siRNAs is a more moderate 2-fold, bringing the ratio of 24:21nt sRNAs to 0.9:1 (0.43:1 in *atnrpd1-3*). The higher level of 24 nt sRNAs in *brnrpd1-2* compared to *atnrpd1-3* suggests that there are more Pol IV-independent 24 nt sRNAs in *B. rapa*. Alternatively, the *brnrpd1-2* mutation might retain some activity, although this is unlikely given the mutation occurs in the Metal A binding site, which is required for activity [43]. There is no change in size profile of sRNAs in the *brnrpd1-1* mutation, suggesting that this allele is functional or hypomorphic.

**Figure 3:**
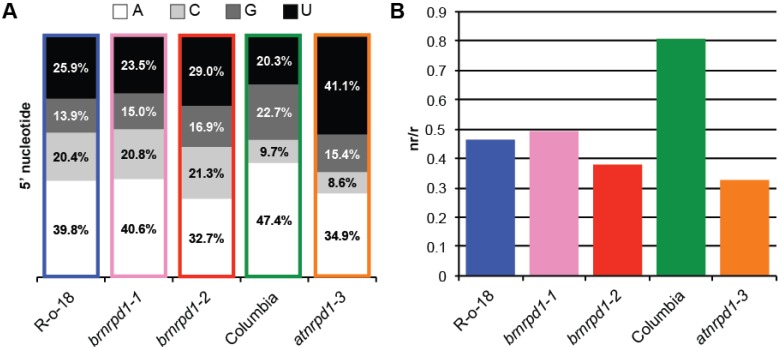
Characteristics of small RNA transcriptomes in *B. rapa* and *Arabidopsis*. (**a**) 5' nucleotide composition of sRNAs. (**b**) Complexity of sRNA populations as measured by the nr/r ratio between sRNA sequences (non-redundant) and reads (redundant). Because the complexity of an sRNA library is inversely proportional to the number of reads in the library, complexity was assessed with 100,000 randomly selected reads from each library.

The 5' nucleotide of a sRNA is also important to direct that molecule into the correct Argonaute effector protein. *Arabidopsis* sRNAs beginning with A are bound by AGO2, AGO4, AGO6, and AGO9, sRNAs beginning with U associate with AGO1, and sRNAs beginning with C are bound by AGO5 [41,42]. Given the deep conservation of microRNA sequence and function, it is likely that these specificities are conserved among plants. In *B. rapa* first nucleotide frequencies are similar to *Arabidopsis*, with fewer A and G 5' nucleotides and correspondingly more C and U (Figure 3A). As expected, loss-of-function *nrpd1* mutations cause a reduction in the level of sRNAs beginning with an A in both species, likely due to a reduction in 24 nt sRNAs associated with AGO4, AGO6, or AGO9. 

Different sRNA classes also display different population complexities. MiRNAs are precisely cleaved from relatively small precursors, resulting in many copies of the exact sRNA sequence. In comparison, p4-siRNAs are randomly cleaved from larger double-stranded precursors, generating a highly diverse mixture of sRNA sequences. The *Arabidopsis* sRNA transcriptome is much more diverse than *B. rapa* and this diversity is due to Pol IV-dependent siRNAs, because complexity drops sharply in the *atnrpd1-3* mutant (Figure 3B). In *brnrpd1-2* complexity is also lower than R-o-18, however it is higher than *atnrpd1-3*, indicating that Pol IV-independent sRNAs are more complex in *B. rapa* than in *Arabidopsis*. 

**Figure 4:**
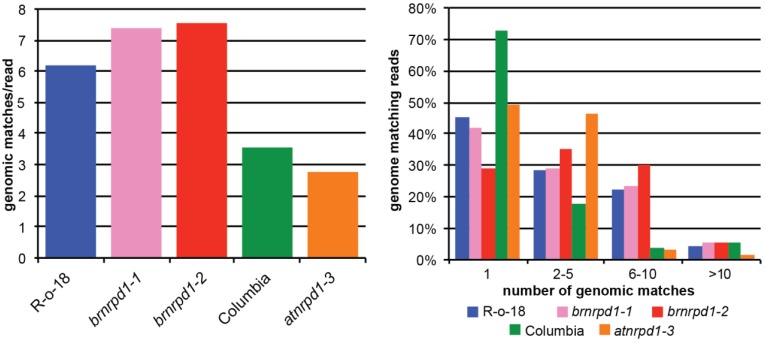
Genomic repetitiveness of small RNA transcriptomes in *B. rapa* and *Arabidopsis*. Average genomic matches per read (**left**) and distribution of genomic repetitiveness (**right**).

Pol IV-dependent siRNAs are frequently generated from repetitive genomic elements such as TEs and therefore have several perfect matches in the genome. Measuring the average number of genomic matches per sRNA is therefore one measure of the repetitive content of the sRNA population. Wild-type *B. rapa* sRNAs match the genome more than 6 times on average, compared to approximately 3.5 times for wild-type *Arabidopsis* sRNAs (Figure 4 left). This difference is due primarily to increases in moderately repetitive sequences (matching the genome 2–10 times), which are more frequent in *B. rapa* than in *Arabidopsis* (Figure 4 right). Reads matching the genome more than 10 times are more abundant in *Arabidopsis* than *B. rapa* and these reads are strongly diminished in *atnrpd1-3* (from 5.5% to 1.5%). Surprisingly, this class is more abundant in the *brnrpd1-2* mutant compared to R-o-18 (5.2% *versus* 4.3%). This suggests that while highly repetitive DNA produces Pol IV-dependent siRNAs in *Arabidopsis*, in *B. rapa* repetitive DNA generates siRNAs through a Pol IV-independent pathway.

### 3.3. Pol IV-Dependent Loci in *Brassica rapa*

**Figure 5:**
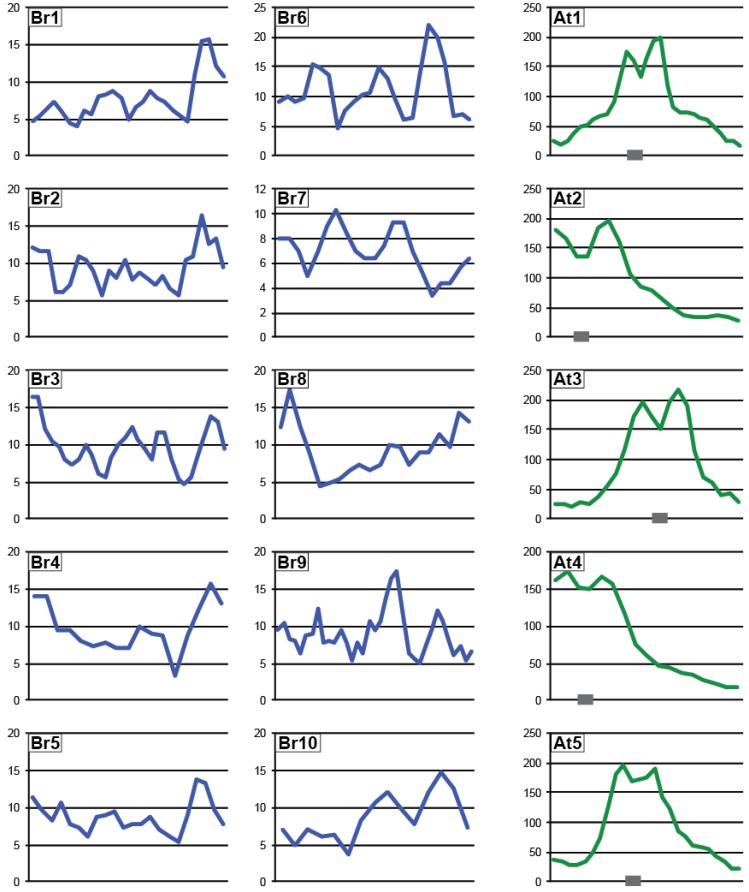
Chromosomal distribution of sRNA-producing loci. Number of siRNA-producing windows in a 1Mb bin (rolling average of 3 bins) across the ten *B. rapa* and 5 *Arabidopsis* chromosomes. Location of the *Arabidopsis* centromeres (as in [44]) are noted with grey boxes.

In *Arabidopsis*, Pol IV-dependent siRNAs are produced from thousands of genomic loci [3,8,9]. To assess the genomic regions producing p4-siRNAs in *B. rapa*, sRNAs were matched to the reference genome and quantified in static 500 base windows. Abundance in each window was normalized to total library size with reads matching to multiple genomic sites counted fractionally at each location and uniquely-mapping sRNAs flagged. Of the 512,523 total windows in the *B. rapa* genome, 2,317 windows (0.45%) had at least one uniquely mapping read and an HNA (Hits Normalized Abundance) ≥15 reads per million, a level of sRNA production that allows confident analysis. Similar investigation of *Arabidopsis* sRNAs identified 10,305 windows (4.3% of 238,296 windows) passing these thresholds. Small RNA loci in *B. rapa* are present across each chromosome, while sRNA loci are most abundant in pericentromeric regions of *Arabidopsis* chromosomes (Figure 5).

**Figure 6:**
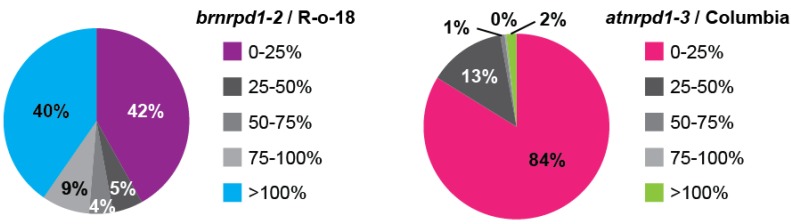
Pol IV-dependent siRNA loci in *B. rapa* and *Arabidopsis.* Small RNA-generating windows were divided into five groups based on the degree of sRNA accumulation in the *brnrpd1-2* or *atnrpd1-3* mutants compared to respective wild types. Pie charts display the proportion of sRNA-generating windows falling into each Pol IV-dependency group (*n* = 2,317 for *B. rapa*, 10,305 for *Arabidopsis*).

To determine the genomic loci producing p4-siRNAs, sRNA-producing windows were categorized based on the HNA in *brnrpd1-2* compared to R-o-18. 42% (971 windows) display strong depletion of sRNAs in *brnrpd1-2*, including 472 windows (20% of sRNA-producing windows) with <5% of R-o-18 HNA. A similar number of windows (935, 40%) show increased sRNA accumulation in the *brnrpd1-2* mutant, although this is mostly likely due to oversampling of unchanged Pol IV-independent sRNAs [3,19,45] (Figure 6). This pattern demonstrates that the *brnrpd1-2* mutation has a strong effect on sRNA biogenesis at specific loci rather than a weak effect at all loci, suggesting it is a null allele. In *Arabidopsis*, 84% (8637) of sRNA-producing windows are Pol IV-dependent and only 179 windows (1.7%) are Pol IV-independent. When the entire genome is considered, a window in *Arabidopsis* is almost 20 times more likely to produced Pol IV-dependent siRNAs than a window in the *B. rapa* genome (3.6% *versus* 0.19%), but a window in the *B. rapa* genome is almost 2.5 times as likely to produce Pol IV-independent sRNAs (0.18% *versus* 0.075%). The increased number of Pol IV-dependent sRNA loci in *Arabidopsis* compared to *B. rapa* might explain the difference in chromosomal distribution, as most of the loci present in the pericentromere of *Arabidopsis* are Pol IV-dependent (Supplementary Figure S2). Although the sRNA size profile in *brnrpd1-1* is very similar to R-o-18, analysis of genomic windows suggests this allele is hypomorphic, due to the correlation between sRNA accumulation in *brnrpd1-1* and *brnrpd1-2* mutants (Supplementary Figure S3).

**Figure 7:**
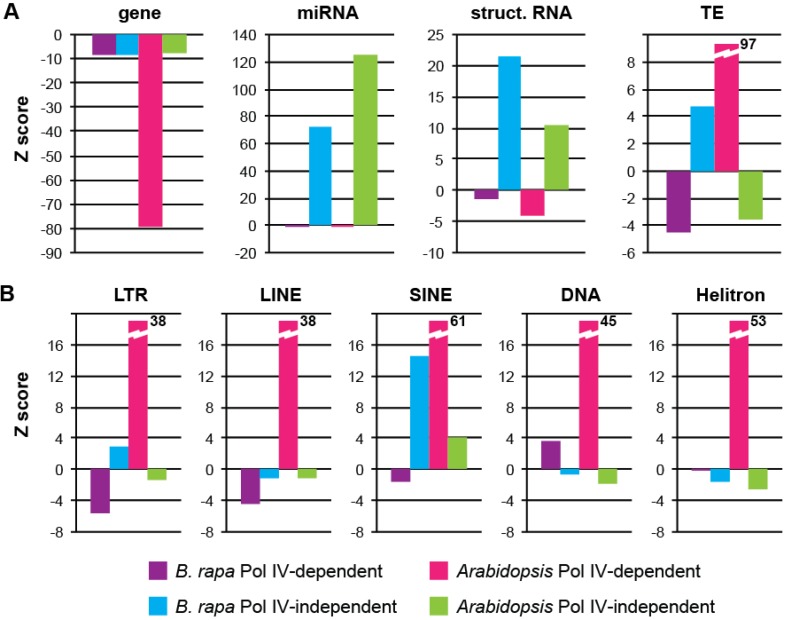
Genomic features at sRNA-generating windows*.* Over- and under-representation of annotation classes for Pol IV-dependent windows (0–25% class) and Pol IV-independent windows (>100% class). (**a**) General genome features. (**b**) Transposable element classes.

Annotations from each window were collected to assess which genomic features are associated with Pol IV-dependent and Pol IV-independent sRNA production in *B. rapa* (Supplementary Figure S4). Genes are moderately underrepresented in both Pol IV-dependent and Pol IV-independent loci, while microRNAs and windows overlapping structural RNA genes (rRNA, tRNA, snRNA) are overrepresented only among Pol IV-independent loci (Figure 7A). Similar patterns are seen at *Arabidopsis* Pol IV-independent and Pol IV-dependent loci, although genes are more highly depleted in Pol IV-dependent loci. Surprisingly, TEs are slightly underrepresented among *B. rapa* Pol IV-dependent loci and slightly over-represented among Pol IV-independent loci. This is opposite of the pattern detected in *Arabidopsis* and predicted by models of Pol IV function.

Further analysis of the types of TEs associated with each group of sRNA loci demonstrates mild but significant depletion of LTR and LINE retrotransposons among *B. rapa* Pol IV-dependent loci, while DNA transposons are slightly overrepresented (Figure 7B). Among Pol IV-independent loci LTR retrotransposons are barely over-represented while SINE elements are significantly overrepresented. In *Arabidopsis*, all categories of TEs display strong overrepresentation specifically at Pol IV-dependent loci. These differences suggest that biogenesis of sRNAs from TEs is distinct in *Brassica* and *Arabidopsis* and that the concordance between p4-siRNA production and TE presence in *Arabidopsis* might not be conserved among all plants. 

## 4. Conclusions

Although *B. rapa* and *Arabidopsis* are close relatives, their sRNA transcriptomes are surprisingly different. In *Arabidopsis*, the Pol IV pathway produces abundant 24 nt sRNAs matching thousands of genomic locations while in *B. rapa*, Pol IV produces a moderate level of 24 nt sRNA from several hundred genomic locations. Pol IV activity is focused on pericentromeric sequences in *Arabidopsis* and is distributed across the chromosomes in *B. rapa*. Although it is possible that the *brnrpd1-2* allele retains some function, the large number of loci that lose all sRNA production and the small number of loci with intermediate sRNA production in *brnprd1-2* make it likely that this is a null mutation. 

Most flowering plants possess more 24 nt than 21 nt sRNAs [19,20,21,22,23,24,25], however the balance between these sizes is unusually high in *Arabidopsis* floral tissue, suggesting that *Arabidopsis* might be an outlier. It is possible that the Pol IV pathway has been recently recruited to pericentromeric TEs in *Arabidopsis* and is hyperactive at these sequences. Abundant p4-siRNA production from the pericentromere would boost the 24:21 nt ratio and might mask “normal” p4-siRNA production from genomic sites distributed across the chromosomes. Alternatively, the floral-specific expression of many Pol IV loci in *Arabidopsis* [46] might be absent in *B. rapa*, resulting in a “vegetative” expression pattern throughout development. Further analysis of sRNA transcriptomes in the *Brassicacea* is needed to unravel these possibilities.

In *Arabidopsis*, Pol IV transcribes all classes of TE [3] and is required for transcriptional silencing of some elements [10,11,47]. Although 13% of identified Pol IV-dependent loci overlap annotated TEs in *B. rapa* (Supplementary Figure S4), TE sequences are not enriched at Pol IV sites compared to the entire genome. Instead, some highly repetitive sequences in *B. rapa* produce Pol IV-independent siRNAs, reminiscent of the RDR2-independent 22 nt siRNAs produced from highly repetitive TE sequences in maize [19]. Twenty-four nt sRNAs from many species are enriched in TE sequences compared to 21 nt sRNAs, however it is unclear whether they are enriched compared to the genome as a whole. Additional analysis of sRNA transcriptomes from sequenced genomes is needed to strengthen the assumption that 24 nt sRNAs are primarily TE in origin. 

Our analysis of a *B. rapa* Pol IV mutant highlights the diverse populations of sRNAs produced in plants and the hazards of drawing models based on a single species. *B. rapa* displays limited Pol IV activity relative to *Arabidopsis*, which might have allowed Pol IV-independent pathways to proliferate. Our research demonstrates that Pol IV-independent 24 nt sRNAs might be as abundant as 21 nt sRNAs in many plant species. It will be interesting to unravel the biogenesis of Pol IV-independent 24 nt sRNAs and discover whether they integrate into AGO4/AGO6/AGO9 and target RNA-directed DNA methylation or participate in other silencing pathways. 

## Supplementary Files

Supplementary File 1 Supplementary (DOCX, 508 KB)
