# Supplementary material for: Pol IV-Dependent siRNA Production is Reduced in Brassica rapa
Source: Biology (Basel). 2013 Sep 30;2(4):1210–23. doi: 10.3390/biology2041210 (PMC4009798; doi:10.3390/biology2041210)

**Table S1.** Brassica orthologs and paralogs of *Arabidopsis* genes.

| At gene | AGI | Syntenic ortholog | Subgenome | Non-syntenic ortholog |
| --- | --- | --- | --- | --- |
| *Pol IV subunits and associated proteins* | | | | |
| NRPD1 | At1g63020 | Bra027611 | MF2 | Bra041010* |
| NRPD2 | At3g23780, At3g18090 | Bra022279, Bra021293 * | LF, MF1 |  |
| NRPE1 | At2g40030 | Bra000162 | MF2 |  |
| NRPD4 | At4g15950 |  |  |  |
| NRPE5 | At3g57080 | Bra007300 | MF1 |  |
| NRPD7 | At3g22900 | Bra023791 | MF1 |  |
| NRPE7 | At4g14660 | Bra0391721 | MF2 |  |
| RDR2 | At4g11130 | Bra035249 | LF |  |
| DCL3 | At3g43920 | Bra019456 | MF2 |  |
| AGO4 | At2g27040 | Bra034318 | MF1 |  |
| AGO6 | At2g32940 | Bra022918 | MF2 | Bra022057 * |
| AGO9 | At5g21150 | Bra002360/Bra002361, Bra020152 | LF, MF2 |  |
| CLSY1 | At3g42670 |  |  | Bra021058 |
| DRD1 | At2g16390 | Bra013077 | MF2 |  |
| DRM2 | At5g14620 | Bra08749, Bra023479 */ Bra023480 * | LF, MF2 |  |
| KTF1 | At5g04290 | Bra009470 | LF |  |
| *Pol II genes* | | | | |
| NRPB1 | At4g35800 | Bra010510 | MF2 |  |
| NRPB2 | At4g21710 | Bra013550, Bra038761, Bra020873/Bra020874 * | LF, MF1, MF2 | Bra020911 |
| NRPB4 | At5g09920 | Bra009073, Bra006039, Bra028609 | LF, MF1, MF2 | Bra039605 * |
| NRPB5 | At3g22320 | Bra031350, Bra001864 | LF, MF2 |  |
| NRPB7 | At5g59180 | Bra002591, Bra006732, Bra020336 | LF, MF1, MF2 | Bra024065 |
| *Other RNA silencing genes* | | | | |
| RDR1 | At1g14790 | Bra026187 | LF |  |
| RDR6 | At3g49500 | Bra029957 | MF1 |  |
| DCL1 | At1g01040 | Bra033293 | LF |  |
| DCL2 | At3g03300 | Bra031999 | LF |  |
| DCL4 | At5g20320 | Bra002293 | LF |  |
| AGO1 | At1g48410 | Bra032254 | MF2 |  |
| AGO2 | At1g31280 | Bra023172 | LF | Bra040815 |
| AGO7 | At1g69440 | Bra003999 | MF2 |  |
| AGO10 | At5g43810 | Bra033698, Bra027505 * | LF, MF2 | Bra014136 |

* Partial gene.

**Figure S1.** Confirmation of putative TILLING mutants. Schematic of the BrNRPD1 genomic region surrounding the *brnrpd1-1* and *brnrpd1-2* mutations with examples of CAPS/dCAPS marker results (M, homozygous mutant; H, heterozygous; W, wild type).


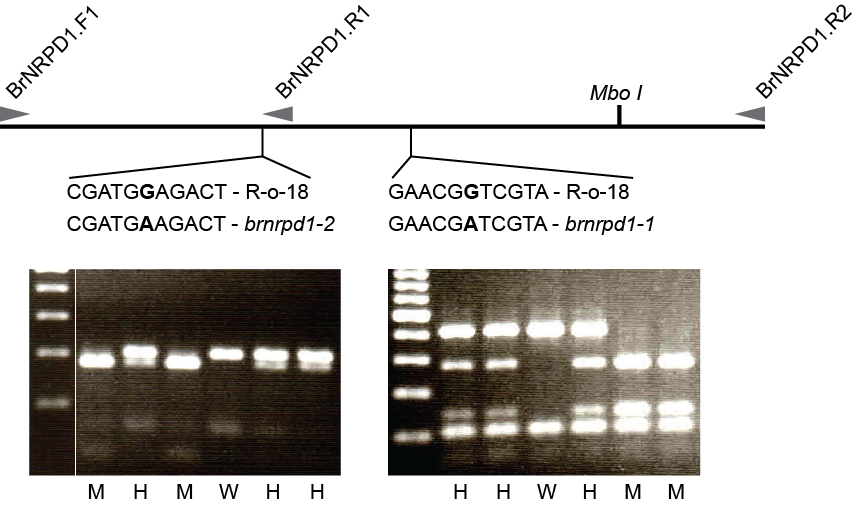


**Figure S2.** Chromosomal distribution of Pol IV-dependent and -independent loci. Number of Pol IV-dependent (0-25) and Pol IV-independent (>100) siRNA-producing windows in a 1Mb bin (rolling average of 3 bins) across the ten *B. rapa* and 5 *Arabidopsis* chromosomes.


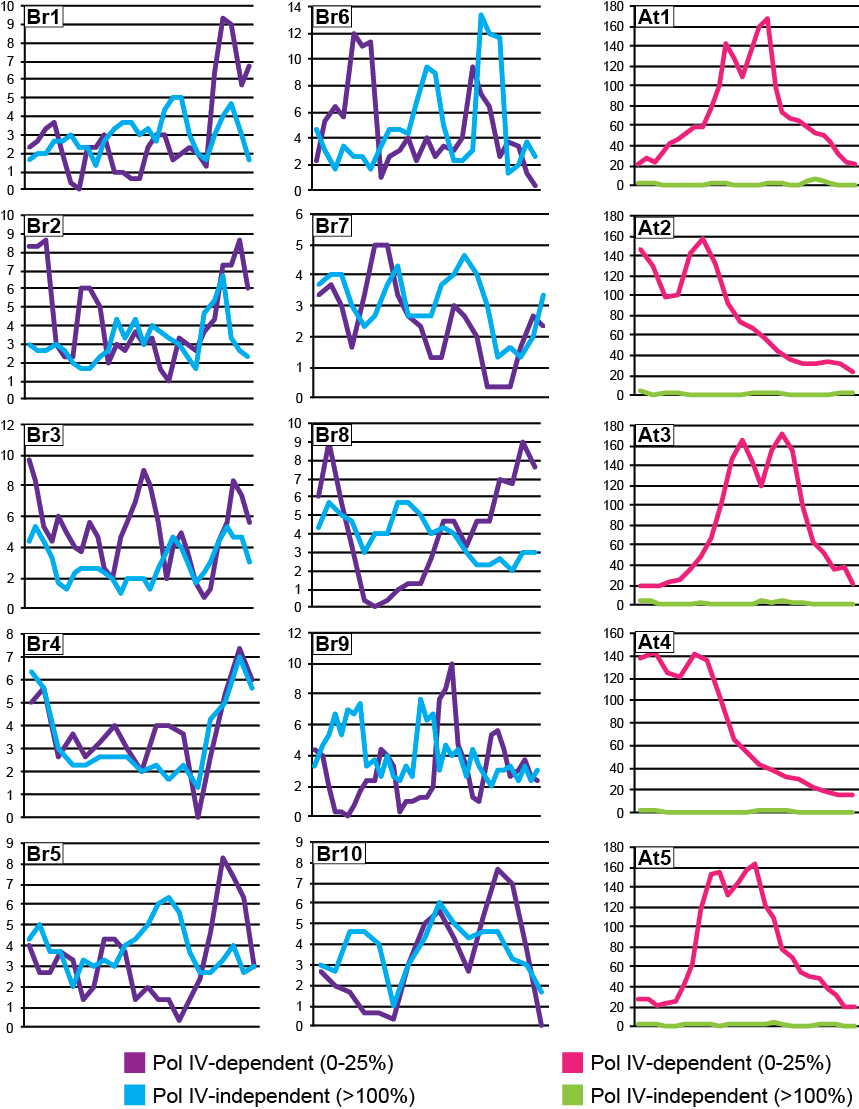


**Figure S3.** Correlation between *brnrpd1-1* and *brnrpd1-2* mutants. Relative abundance (HNA *brnrpd1-1*/HNA R-o-18) was calculated for each sRNA-producing window and averaged within the Pol IV-dependency groups established in Figure 4A.


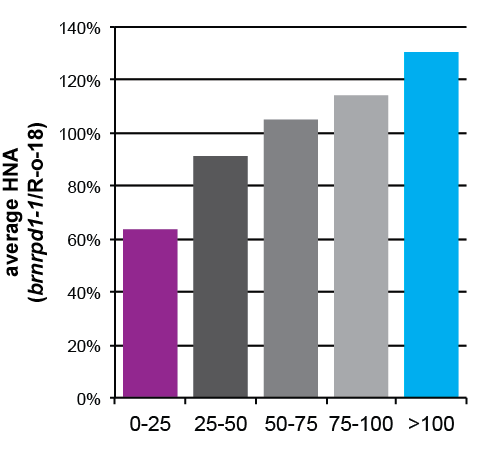


**Figure S4.** Genomic loci producing sRNAs. Genomic annotations overlapping
sRNA-producing windows in *B. rapa*. Windows overlapping more than one genomic feature were assigned a single annotation based on miRNA > t/r/snRNA > TE > gene.


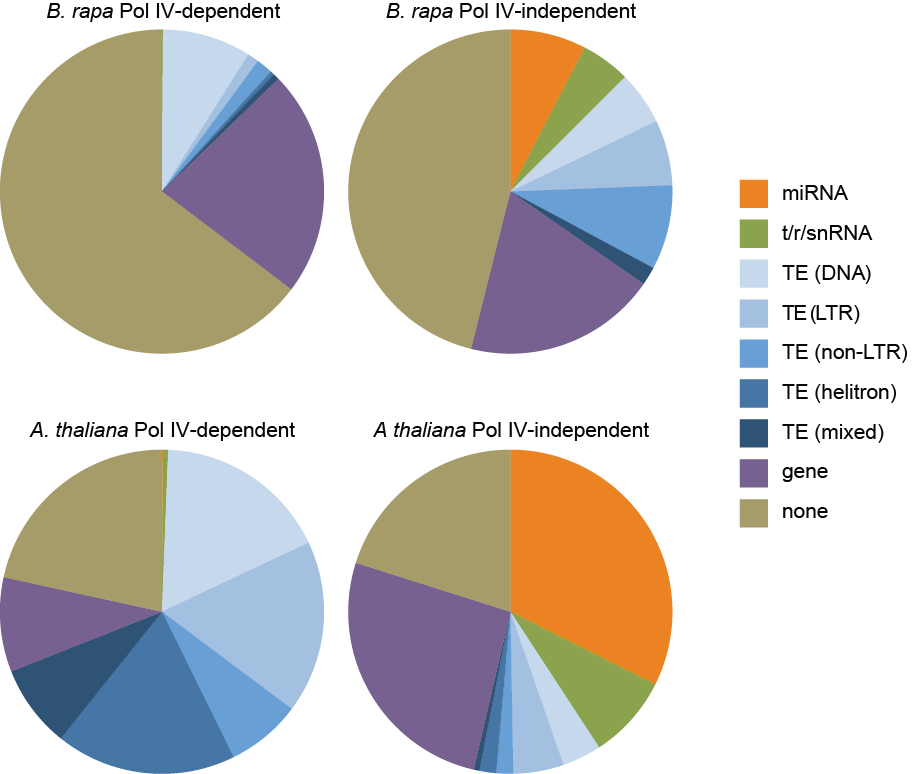

Supplement: Supplementary File 1 — Supplementary (DOCX, 508 KB) [file biology-02-01210-s001.docx]
